# Salirasib inhibits the growth of hepatocarcinoma cell lines in vitro and tumor growth in vivo through ras and mTOR inhibition

**DOI:** 10.1186/1476-4598-9-256

**Published:** 2010-09-22

**Authors:** Nicolas Charette, Christine De Saeger, Valérie Lannoy, Yves Horsmans, Isabelle Leclercq, Peter Stärkel

**Affiliations:** 1 Laboratory of Gastroenterology, Institut de Recherche Expérimentale et Clinique, Université Catholique de Louvain, 1200 Brussels, Belgium; 2 Department of Gastroenterology, Cliniques Universitaires Saint-Luc, 1200 Brussels, Belgium

## Abstract

**Background:**

Dysregulation of epidermal growth factor and insulin-like growth factor signaling play important roles in human hepatocellular carcinoma (HCC), leading to frequent activation of their downstream targets, the ras/raf/extracellular signal-regulated kinase (ERK) and the phosphoinositide 3-kinase (PI3K)/Akt/mammalian Target of Rapamycin (mTOR) pathways. Salirasib is an S-prenyl-cysteine analog that has been shown to block ras and/or mTOR activation in several non hepatic tumor cell lines. We investigated *in vitro *the effect of salirasib on cell growth as well as its mechanism of action in human hepatoma cell lines (HepG2, Huh7, and Hep3B) and its *in vivo *effect in a subcutaneous xenograft model with HepG2 cells.

**Results:**

Salirasib induced a time and dose dependent growth inhibition in hepatocarcinoma cells through inhibition of proliferation and partially through induction of apoptosis. A 50 percent reduction in cell growth was obtained in all three cell lines at a dose of 150 μM when they were cultured with serum. By contrast, salirasib was more potent at reducing cell growth after stimulation with EGF or IGF2 under serum-free conditions, with an IC_50 _ranging from 60 μM to 85 μM. The drug-induced anti-proliferative effect was associated with downregulation of cyclin A and to a lesser extent of cyclin D1, and upregulation of p21 and p27. Apoptosis induction was related to a global pro-apoptotic balance with caspase 3 activation, cytochrome c release, death receptor upregulation, and a reduced mRNA expression of the apoptosis inhibitors cFLIP and survivin. These effects were associated with ras downregulation and mTOR inhibition, without reduction of ERK and Akt activation. *In vivo*, salirasib reduced tumour growth from day 5 onwards. After 12 days of treatment, mean tumor weight was diminished by 56 percent in the treated animals.

**Conclusions:**

Our results show for the first time that salirasib inhibits the growth of human hepatoma cell lines through inhibition of proliferation and induction of apoptosis, which is associated with ras and mTOR inhibition. The therapeutic potential of salirasib in human HCC was further confirmed in a subcutaneous xenograft model.

## Background

Hepatocellular carcinoma (HCC) is the fifth most common cancer worldwide, and the third leading cause of cancer related mortality. Its incidence has more than doubled during the last two decades in the western world, where it is the fastest growing cause of cancer related death [1]. Despite the magnitude of the problem, existing therapies are of limited efficacy. No more than 30% of the patients are eligible for curative treatment, and recurrence is a frequent issue affecting up to 70% of the patients after tumor ablation. Moreover, due to underlying cirrhosis, systemic therapy with classical cytotoxic drugs is poorly tolerated and ineffective [2]. Accordingly, new therapeutic approaches for this disease are eagerly awaited.

Several growth factor signaling pathways are dysregulated in hepatocarcinogenesis [3]. In particular, altered intracellular signaling elicited by epidermal growth factor (EGF), insulin-like growth factor (IGF) and Vascular Endothelial Growth Factor have been involved in the pathogenesis of HCC. Hence, inhibitors of their receptors are under intense investigation. While anti-IGF receptor (IGF-1R)-based therapies are currently studied in preclinical and early clinical trials, inhibition of the EGF receptor (EGFR) by either tyrosine kinase inhibitors or monoclonal antibodies has shown limited efficacy in several phase II studies in HCC [4]. In non hepatic epithelial tumor cell lines, inhibition of EGFR or IGF-1R individually promotes activation of the reciprocal receptor [5] and IGF-2 overexpression has been involved in the resistance of HCC to EGFR inhibition in a rat model [6]. Treatment interfering with both receptors could thus represent a better strategy to treat this disease. Alternatively, targeting one or several of their downstream signaling pathways could be an elegant way to block growth factor signaling. Among those, both ras-raf-MEK-ERK and PI3K-Akt-mTOR pathways are activated upon EGFR and IGF-1R stimulation. While ras activation upon EGFR stimulation induces PI3K activation [7], IGF-1R is able to activate the PI3K-Akt-mTOR pathway independently of ras [8].

Ras activation has been shown to be an ubiquitous and early event in human HCC [9], whereas mTOR activation is present in half of the cases [10]. Downstream receptor signaling inactivation has proved its efficacy as demonstrated by the results of the SHARP trial evaluating sorafenib, a multikinase inhibitor targeting the VEGFR and PDGFR kinases as well as raf, in advanced HCC. However, it only leads to a modest increase in median overall survival of 3 months [11], highlighting the need for the development of new and more effective targeted therapies for HCC.

Salirasib (Farnesylthiosalicylic acid, FTS) is a S-farnesyl cysteine analog that affects docking of active GTP-bound ras in the cell membrane by competing with ras for its membrane anchorage sites and consequently inhibits ras-dependent cell growth [12]. In cell lines, this leads to an accelerated degradation of cytosolic ras and a decrease in the total amount of cellular ras [13]. This mode of action affecting all ras isoforms differentiates salirasib from farnesyltransferase inhibitors, which fail to block K-ras and N-ras activity because they undergo geranylgeranylation following treatment with those molecules [7]. In addition, salirasib has also been shown to directly inhibit mTOR complex 1 activity by disruption of the mTOR-raptor complex [14]. It exhibits anti-tumoral effects in several non-liver cancer cell lines [15] and has recently been evaluated in a phase 1 study in patients with solid non hepatic tumors, showing that it was well tolerated [16]. Targeting both ras and mTOR, along with a good tolerance in patients, make salirasib a good candidate for HCC therapy.

Previous work of our team has shown that high dose salirasib blocks hepatocytes proliferation in vivo in rats after partial hepatectomy [17]. This inhibitory effect was at least partially mediated by inhibition of ERK phosphorylation. More recently, we have shown that salirasib administration prevents liver tumor development in a model of diethylnitrosamine-induced hepatocarcinogenesis in rats [18].

The aims of the present study are to evaluate the efficacy of salirasib in human HCC cell lines, and to understand its underlying molecular mechanisms of action in these particular cells thereby providing a rationale for testing it as a novel anti-cancer treatment in HCC clinical trials.

## Methods

### Compounds

Salirasib was kindly provided by Concordia Pharmaceuticals (Fort Lauderdale, Florida, USA). Unless stated otherwise, all cell culture reagents and growth factors were purchased from Invitrogen (Brussels, Belgium). Antibodies were from Santa Cruz (Santa Cruz, California, USA), Millipore (Billerica, Massachusetts, USA), Cell Signaling (Danvers, Massachusetts, USA), BD Biosciences (Erembodegem, Belgium), or Sigma-Aldrich (Bornem, Belgium).

### Cell culture

**Figure 1:**
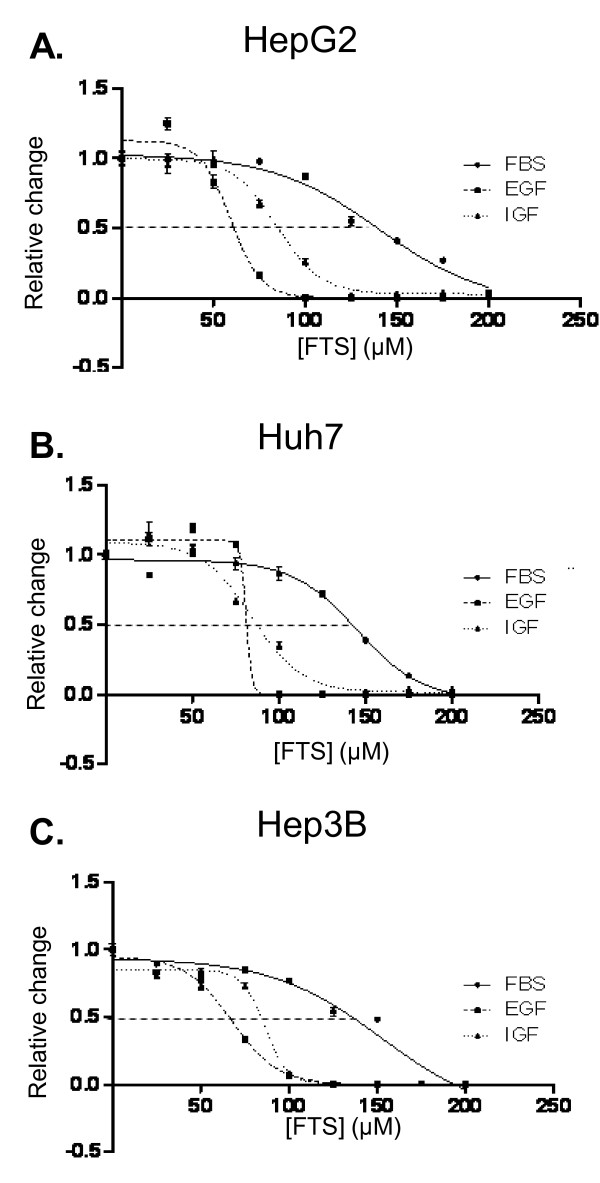
**Salirasib induces a dose- and time-dependent decrease in HCC cells viability**. A-C. HepG2 (A), Huh7 (B), and Hep3B (C) cells were plated in 96-wells plates and incubated with various doses of salirasib for 3 days (n = 8 at each dose). Cell viability was assessed by WST-1 expression and IC_50 _was determined using nonlinear regression analysis. Identical experiments were performed in FBS-cultured cells as well as in EGF- and IGF2-stimulated cells. Data are presented as mean ± SEM.

HepG2, Huh7 and Hep3B were obtained from European Collection of Cell Culture (Salisbury, United Kingdom) and cultured in Dulbecco's modified eagle medium (HepG2 and Huh7) or minimum essential medium containing Earle's salt (Hep3B) supplemented with 10% fetal bovine serum (FBS), 1% streptomycin and penicillin, 1% non essential amino-acid, plus 1% sodium pyruvate for HepG2, in 5% CO2 at 37°C. Medium was renewed once a day. Cells were seeded in 6-well plates (cell count, cell cycle analysis, RNA isolation) or in 96-well plates (WST-1, BrdU, caspase, and LDH assays) at a density of 1-5 × 10^5 ^and 5 × 10^3^cells per well, respectively. For protein preparation, cells were plated in 10 cm Petri dishes at a density of 1.5 × 10^6 ^(4.5 × 10^6 ^for ras pull-down assays). Cells were allowed to adhere overnight. Thereafter, they were incubated in medium supplemented with 0.1% dimethylsulfoxyde (DMSO, vehicle) or salirasib (50 μM, 100 μM or 150 μM) for various durations (2 hours to 7 days). For IC_50 _determination, salirasib concentrations ranging from 25 μM to 200 μM were used. Analyses of cell cycle, RNA and protein were performed in cells exposed to DMSO or 150 μM salirasib during 24 h or 48 h (and 72 hours for cell cycle), for this concentration corresponded to IC_50 _in all 3 tested cell lines (figure 1A-C).

For growth factor simulation, cells were serum starved overnight. EGF (50 ng/ml) or IGF2 (75 ng/ml) were added to serum-free medium supplemented with 0.1% bovine serum albumin (BSA) and cells were stimulated for 2 minutes (ras pull-down assays), 10 minutes (signaling pathway studies), 24 hours (cell proliferation studies) or 3 days (dose-response studies) in the presence of salirasib or DMSO.

All experiments were repeated at least twice on separate days. The total n used for statistical analysis was 6 or 8 per treatment group.

### Growth inhibition studies

For time dependent response studies, cells were harvested with 0.05% Trypsin-EDTA daily for 1 to 7 days and counted under the microscope using the Trypan blue exclusion method.

For dose response studies, cells were incubated in medium supplemented with salirasib or DMSO for 3 days. Cell viability was determined using a colorimetric WST-1 assay (Roche, Vilvoorde, Belgium) according to the manufacturer's instructions. The IC50 value, at which 50% of the cell growth is inhibited compared with DMSO control, was calculated by nonlinear regression analysis using GraphPad Prism software (San Diego, California, USA).

### Determination of DNA synthesis

DNA synthesis was assessed after 1 and 2 days of treatment by a colorimetric Bromodeoxyuridine (BrdU) assay (Roche) according to the manufacturer's instructions. BrdU was added for the last 4 h of the experiment.

### Cell cycle analysis

Cell cycle was analyzed after 1, 2 and 3 days of treatment. Briefly, cells were harvested by trypsinization and washed with PBS. They were fixed in ice-cold ethanol, washed, resuspended in PBS and treated with RNase A (Sigma-Aldrich). Finally, cells were stained with propidium iodide (Sigma-Aldrich) and analyzed by flow cytometry (BD FACSCalibur, BD Biosciences). DNA content was quantified using CellQuest Pro software (BD Biosciences).

### Determination of caspase-3/7 activity and LDH release

Caspase activity and LDH release were assessed after 24 h of treatment using the Caspase-Glo 3/7 assay (Promega, Leiden, Netherlands) and the Cytotoxicity Detection KitPlus (Roche), respectively, according to the manufacturer's instructions.

### Western blotting

Cells were harvested in ice-cold lysis buffer [0.1% Triton X-100, 50 mM HEPES (pH 7.5), 150 mM NaCl, 10% (v/v) glycerol, 1.5 mM MgCl2, 1 mM dithiothreitol, 1 mM sodium fluoride, 0.1 mM sodium orthovanadate, 1 mM phenylmethylsulfonyl fluoride, 2 μg/ml leupeptin and aprotinin]. Equal amounts of proteins, determined by a BCA protein assay (Pierce, Rockford, Illinois, USA), were separated by SDS/PAGE and transferred on to polyvinylidene fluoride membranes according to standard techniques. Primary and secondary antibodies as well as working conditions are listed in Additional file 1, Table S1. Membranes were revealed with the 'Western Lightning Chemiluminescent Reagent Plus' (Perkin Elmer, Boston, MA, USA) detection system and immunoreactive proteins were quantified by densitometry using the Gel Doc™ XR System 170-8170 device and software (Bio-Rad, Nazareth, Belgium) and normalized to their respective loading controls, HSP90 or β-actin. In order to compare the independent experiments, data were expressed as relative change compared to the control (DMSO) group which was arbitrarily set at 1 for each experiment.

### Ras pull down assay

Cells were harvested in ice-cold Mg2+ lysis/wash buffer (Millipore, Cat.# 20-168) supplemented with 1 mM sodium fluoride, 0.1 mM sodium orthovanadate, 1 mM phenylmethylsulfonyl fluoride, 2 μg/ml leupeptin and aprotinin. Lysates were centrifuged at 4°C and supernatant containing 500 μg of proteins was mixed with 10 μl of Raf1-ras-binding-domain agarose beads (Millipore, Cat.# 14-278), rotated at 4°C for 1 hour, washed three times with lysis/wash buffer, boiled for 5 minutes in Laemmli buffer under reducing conditions, and separated by SDS/PAGE. Activated ras protein was then revealed by immunoblotting with a pan-ras antibody (Millipore, Cat.# 05-516).

### Reverse transcription and quantitative PCR

Cells were harvested in Trizol Reagent (Invitrogen) for RNA extraction. RNA was reverse transcribed and subjected to quantitative PCR with the StepOnePlus™ Real Time PCR System (Applied Biosystems, Lennik, Belgium) using SYBRgreen. Primers were designed using the Primer Express™ design software (Applied Biosystems, Lennik, Belgium) and sequences are presented in Additional file 2, Table S2. Quantification was obtained according to the ∆∆CT method [19]. The final result of each sample was normalized to its respective Ribosomal protein L19 (RPL19, internal standard) value.

### Tumor xenograft experiments

Six week old female athymic NMRI nu/nu mice (Elevage Janvier, Le Genest-St-Isle, France) were housed in filter-topped cages and received food and water *ad libitum*. Tumors were generated by subcutaneous injection into the right lower flank with 5 × 10^6 ^HepG2 cells suspended in 100 μl PBS in 12 mice. Two weeks after cell inoculation, when palpable tumours were established, mice were separated into salirasib-treated (n = 6) and control group (n = 4). Two animals did not develop tumours at that time point and had to be excluded from the study. They received daily i.p. injections of 10 mg/kg salirasib or a similar volume of vehicle solution (PBS containing 2.5% v/v ethanol, pH 8.0) for 12 days. Tumor dimensions were recorded three times per week with a digital calliper starting with the first day of treatment. Tumor volumes were estimated as follows: V (mm³) = (length × width²)/2. Tumour weights were recorded at the time of sacrifice in order to evaluate treatment response. The animals were handled according to the guidelines for humane care for laboratory animals established by the Université Catholique de Louvain in accordance with EU regulation. The study protocol was approved by the local ethics committee.

### Statistical analysis

Results are expressed as relative change compared with DMSO controls and are given as the mean ± SEM. The statistical differences between groups were tested using a two-tailed Student's *t *test. Statistical significance was assumed for *P *values <0.05.

## Results

### Salirasib induces a dose- and time-dependent decrease of cell growth in HCC cells

Incubation of FBS-cultured cells with salirasib for 3 days resulted in a dose-dependent growth inhibition with an IC_50 _of 149 μM in HepG2, 145 μM in Huh7, and 153 μM in Hep3B (figure 1A-C). As FBS is a cocktail of growth factors and cytokines recruiting multiple receptors, we hypothesized that salirasib would be more effective in reducing cell growth in serum starved cells that were selectively exposed to EGF or IGF2 only. Indeed, we observed that salirasib treatment elicited a dose-dependent decrease in cell viability in all 3 cell lines that was more pronounced in both EGF- and IGF2-stimulated cells than in FBS-stimulated cells. Respectively, IC_50 _in EGF- and IGF2- stimulated cells decreased to 59 μM and 85 μM for HepG2, to 81 μM and 85 μM for Huh7, and to 67 μM and 86 μM for Hep3B (figure 1A-C).

In time-course experiments with FBS-cultured cells, we found that 150 μM salirasib led to a statistically significant reduction in cell number already after 24 hours of treatment in all three cell lines, while 3 and 4 days were necessary to obtain a significant reduction in cell number in cells exposed to 100 μM and 50 μM salirasib, respectively (data not shown). After 7 days, cell counts were reduced to 31% of controls in Hep3B cells treated with 50 μM salirasib and to 5% of controls when they were exposed to 100 μM salirasib. In HepG2 cells, cell counts dropped to 54% and 34% of controls when treated with 50 μM and 100 μM salirasib, respectively. In Huh7 cells, the same concentrations of salirasib decreased cell numbers to 70% and 52% of untreated cells, respectively. In the three tested cell lines, no more viable cells were present when exposed to 150 μM salirasib for one week (table 1).

**Table 1:** Salirasib induced decrease in cell number after 7 days of treatment

		Salirasib treatment		
		
		50 μM	100 μM	150 μM
	**HepG2 **(S.E.M.)	54%*** (±5%)	34% *** (±3%)	2% *** (±0%)
	
**Cell line**	**Huh7 **(S.E.M.)	70%** (±6%)	52%*** (±3%)	1%*** (±0%)
	
	**Hep3B **(S.E.M.)	31%*** (±3%)	5%*** (±0%)	0%***

Data (means ± S.E.M.) are expressed as relative change compared with the control group (DMSO) ** *P *< 0.01 and *** *P *< 0.001

### Salirasib reduces cell proliferation through modulation of cell cycle effectors and inhibitors

**Figure 2:**
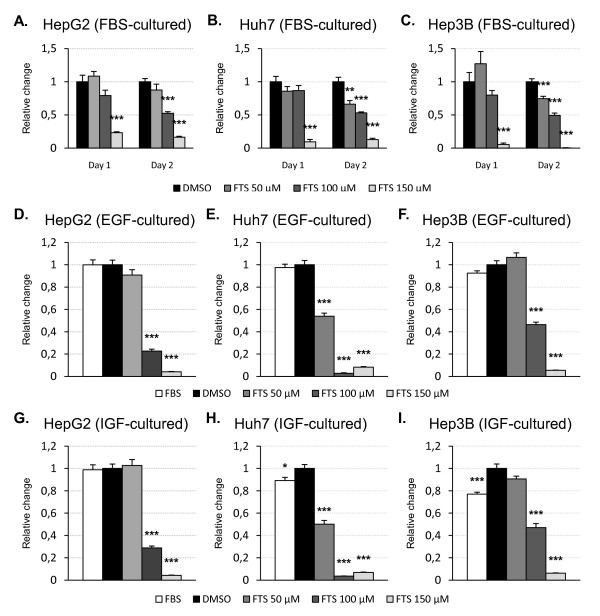
**Salirasib inhibits HCC cells proliferation**. HepG2 (left column), Huh7 (central column), and Hep3B (right column) cells were seeded in 96-wells plates and incubated with DMSO (control) or the indicated doses of salirasib (n = 8 at each dose and time-point). Identical experiments were performed in FBS-cultured cells (A-C), in EGF- (D-F) and IGF2-stimulated (G-I) cells. BrdU incorporation was assessed after 1 (FBS, EGF, IGF2) or 2 days (FBS only) treatment with salirasib. In the growth factor stimulation experiments, a FBS group - consisting of FBS-cultured untreated cells - was included as a positive control. Data are presented as mean ± SEM. * *P *< 0.05, ** *P *< 0.01 and *** *P *< 0.001 in treated groups versus control group.

We next assessed the impact of salirasib on cell proliferation by measuring BrdU incorporation. We observed a time- and dose-dependent decrease in DNA synthesis in all tested cell lines (figure 2A-C), reflecting a reduced cell proliferation. After 24 hours of treatment in FBS-incubated cells, reduction in cell proliferation was only seen in cells exposed to 150 μM salirasib. After 48 hours however, a significant decrease in BrdU incorporation was present at 100 μM in all the tested cell lines and to a lesser extent at 50 μM in Huh7 and Hep3B cells. Inhibition of proliferation was further investigated in EGF- and IGF2-stimulated cells. By contrast to cells incubated with FBS, reduction in BrdU incorporation occurred earlier and at a lower concentration of salirasib in growth factor stimulated cells. Already after 24 hours of treatment, 100 μM salirasib markedly decreased EGF- (figure 2D-F) and IGF2-induced (figure 2G-I) DNA synthesis in HepG2 and Hep3B cells. In Huh7 cells, significant inhibition was even apparent at 50 μM.

K-ras activation is known to regulate cell cycle progression through interference with cyclins and cell cycle inhibitors [20], whereas salirasib has been shown to up-regulate p53 and p21 [21]. The levels of cyclin A, cyclin D1, cyclin E, Cdk2, Cdk4, p27 and p53 were thus evaluated by Western blot analysis, and expression of p21 was assessed by quantitative PCR.

**Figure 3:**
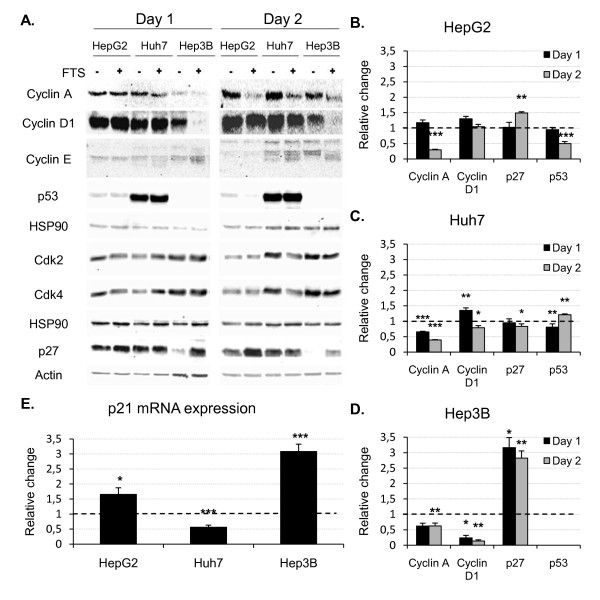
**Salirasib modulates the expression of cell cycle effectors and inhibitors**. A. Representative Western blots after one and two days of treatment in the 3 cell lines. B-D. Quantification of the principal changes in protein expression by densitometry analysis (n = 6 per group) in HepG2 (B), Huh7 (C), and Hep3B (D) cells. E. Quantification of p21 mRNA expression by quantitative PCR after one day of treatment (n = 6 per group). Data are expressed as relative change versus control (DMSO) set arbitrarily at 1 (dotted line) and are presented as mean ± SEM. * *P *< 0.05, ** *P *< 0.01 and *** *P *< 0.001 in treated groups versus control group.

Compared with untreated controls, salirasib induced no significant changes in cyclin E and Cdk2 expression. Cdk4 expression was down-regulated after 2 days of treatment only in Huh7 cells (figure 3A). The most prominent changes in expression of cell cycle effectors were observed for cyclin A and cyclin D1 (figure 3A-D). After 48 hours of treatment, we observed a significant down-regulation of cyclin A in all tested cell lines. Moreover, a significant decrease was already seen in Huh7 cells after 24 hours of treatment, as well as in Hep3B cells, however without reaching statistical significance in the latter cell line (*P *= 0.067). Cyclin D1 was blunted in Hep3B cells as from 24 hours of treatment onwards. A slight but significant reduction was also observed in Huh7 cells after 48 hours, while salirasib did not modify cyclin D1 expression in HepG2 cells.

Expression of the cell cycle inhibitors p27 and p21 was increased by salirasib in HepG2 and Hep3B cells, while p27 remained unchanged and p21 decreased in Huh7 cells (figure 3A-E). p53 expression was markedly down-regulated after 2 days of treatment in HepG2 cells (p53 wild-type). By contrast, the strong basal expression seen in the p53-mutated Huh7 cell line was not modified by salirasib (figure 3A-C). As expected, p53 immunoreactivity was absent in the p53-null Hep3B cell line (figure 3A).

Since our results suggested that salirasib might interfere with the cell cycle, we assessed cell cycle distribution by flow cytometry. Salirasib elicited an increase of the percentage of cells in G0/G1 phase and a concomitant decrease of the percentage of cells in S and G2/M phases (Additional file 3, figure S1). Those changes were already statistically significant after 1 day in Huh7 and after 2 days in HepG2, but only after 3 days in Hep3B cells (data not shown). After 3 days of treatment, 61% of HepG2 cells in the control group were in G0/G1 phase, 16% in S phase and 22% in G2/M phases. By contrast, the percentage of cells in G0/G1 phase increased to 68%, whereas it decreased to 12% and 18% for S and G2/M phases, respectively, in salirasib treated cells. In Huh7 cells, the percentage of cells in G0/G1 phase rose from 49 to 54 after three days of treatment. Concomitantly, the proportion of cells in S phase dropped from 26% to 16%, and that of cells in G2/M phases from 23% to 15%. In Hep3B cells, the proportion of cells in G0/G1, S and G2/M phases was 54%, 12% and 28%, respectively, in control cells and changed to 57%, 10%, and 27%, respectively, in salirasib treated cells. Additionally, salirasib induced an increase in the percentage of sub-G0 cells from 2% to 14% in Huh7 and from 5% to 8% in Hep3B cells.

### Salirasib induces apoptosis in HepG2 and Hep3B cells

**Figure 4:**
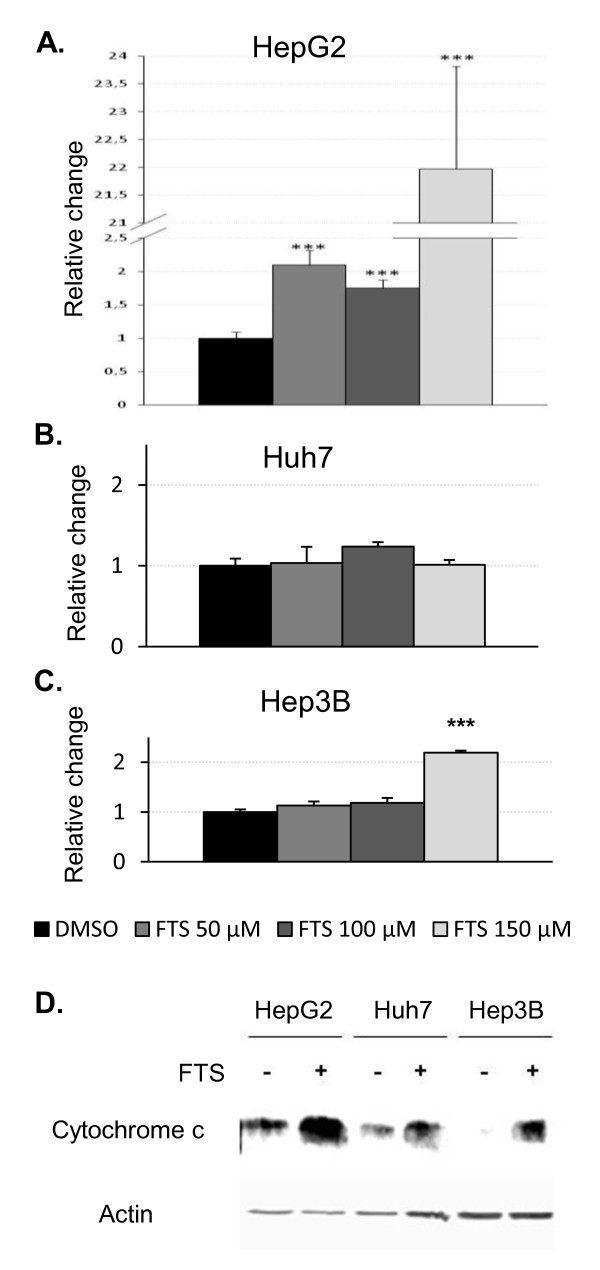
**Salirasib induces apoptosis in HepG2 and Hep3B cells**. A-C. Caspase activity assay performed after 24 hours of treatment with the indicated doses of salirasib in HepG2 (A), Huh7 (B), and Hep3B (C). D. Representative Western blots of three independent experiments of cytochrome c after 24 hours of treatment. Data are expressed as relative change versus control (DMSO) set arbitrarily at 1 and are presented as mean ± SEM. * *P *< 0.05, ** *P *< 0.01 and *** *P *< 0.001 in treated groups versus control group.

As caspase-3 and -7 are the principal effector caspases committing cells to apoptosis [22], we studied their activity upon salirasib treatment in FBS-cultured cells. After 24 hours, it induced a marked increase of caspase-3/7 activity in HepG2 cells (up to 22-fold increase at 150 μM) and a more modest but significant increase in Hep3B cells (2.2 fold increase at 150 μM) (figure 4A, C). Caspase-3/7 was not activated in Huh7 cells (figure 4B). Apoptosis induction was further substantiated by an increase cytochrome c expression detected by western blot analysis (Figure 4D) in HepG2 and Hep3B but not in Huh7 cells, pointing to a possible involvement of the mitochondrial apoptotic pathway. At the same time-point, no LDH activity could be detected in the culture medium of any of the three tested cell lines whether treated or not with salirasib (data not shown).

**Figure 5:**
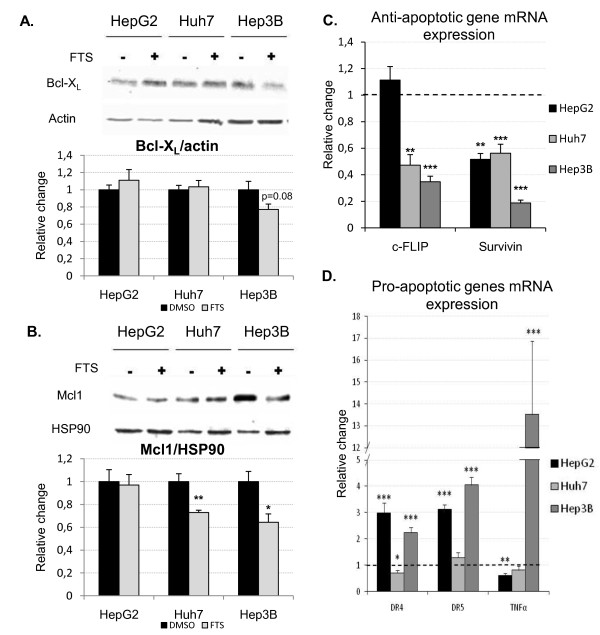
**Modulation of apoptosis effectors and inhibitors by salirasib**. A, B. Representative Western blot of Bcl-X_L _(A) and Mcl-1 (B) after one day of treatment. Western blot of the independent experiments were quantified by densitometry and then normalized to their respective β-actin (Bcl-X_L_) or HSP90 (Mcl-1) (n = 6 per group). Data are finally expressed as relative change compared to the control (DMSO) group arbitrarily set at 1. C, D. Quantification of anti-apoptotic genes (C) and pro-apoptotic genes (D) mRNA expression by quantitative PCR after one day of treatment with salirasib (n = 6 per group). Data of the independent experiments were first normalized to RPL19 (internal standard) and then expressed as relative change versus control (DMSO) set arbitrarily at 1 (dotted line) and are presented as mean ± SEM. * *P *< 0.05, ** *P *< 0.01 and *** *P *< 0.001 in treated groups versus control group.

As our results suggest activation of the intrinsic apoptotic pathway, we studied the expression of Mcl1, Bcl-X_L_, and survivin all of which inhibit this pathway, by Western blot or quantitative PCR. Among the anti-apoptotic members of the Bcl2 family shown to be modified in HCC [23], salirasib significantly reduced Mcl1 expression in Huh7 and Hep3B but not in HepG2 cells, while Bcl-XL levels remained unchanged upon treatment in the three tested cell lines (figure 5A-B). The caspase-3, -7, and -9 inhibitor survivin was strongly repressed in all treated cell lines compared to control (figure 5C).

In addition, since we have previously shown that salirasib induced apoptosis in preneoplastic liver lesions in a rat model of HCC in vivo through activation of the extrinsic apoptotic pathway [18], we studied expression of cellular FLICE-like inhibitory protein (cFLIP), TNF-related apoptosis inducing ligand (TRAIL)-receptor 1 (DR4), TRAIL-receptor 2 (DR5), tumor necrosis factor (TNF)-α, and Fas by quantitative PCR in our human HCC cell lines. The caspase-8 inhibitor c-FLIP was downregulated in Huh7 and Hep3B, but not in HepG2 cells (figure 5C). Expression of the pro-apoptotic TRAIL-receptor DR4 and DR5 mRNA levels were upregulated upon treatment in HepG2 and Hep3B, but not in Huh7 cells (figure 5D). Salirasib treatment elicited a dramatic increase in TNFα mRNA expression in Hep3B cells, while it remained unchanged in Huh7 and was downregulated in HepG2 cells (figure 5D). Finally, Fas expression was increased upon treatment in HepG2 (1.5 fold compared to control, P < 0.05). As Huh7 and Hep3B cells are known to be Fas-deficient, we did not evaluate it in those cell lines. Altogether our results suggest that salirasib induce a pro-apoptotic phenotype with some differences among the 3 cell lines (table 2).

**Table 2:** Summary of apoptosis-related changes

	HepG2	Huh7	Hep3B
Caspase-3/7	↑↑↑	--	↑↑

Cytochrome c	↑ (SA)	↑ (SA)	↑ (SA)

**Mcl1**	--	↓	↓

**Bcl-X**_**L**_	--	--	--

**Survivin**	↓	↓	↓↓

**cFLIP**	--	↓↓	↓↓

DR4	↑↑	--	↑↑

DR5	↑↑	--	↑↑

TNFα	↓	--	↑↑↑

Fas	↑	NE	NE

--: no change; ↑(increase of 1-2x over controls)/↑↑(2-5x)/↑↑↑(>5x); ↓(reduction <2x compared to controls)/↓↓(reduction >2x compared to control); NE: not expressed; SA: subjective appraisal. Bold characters: anti-apoptotic. Plain characters: pro-apoptotic.

### Salirasib reduces ras expression and activation in HCC cells

**Figure 6:**
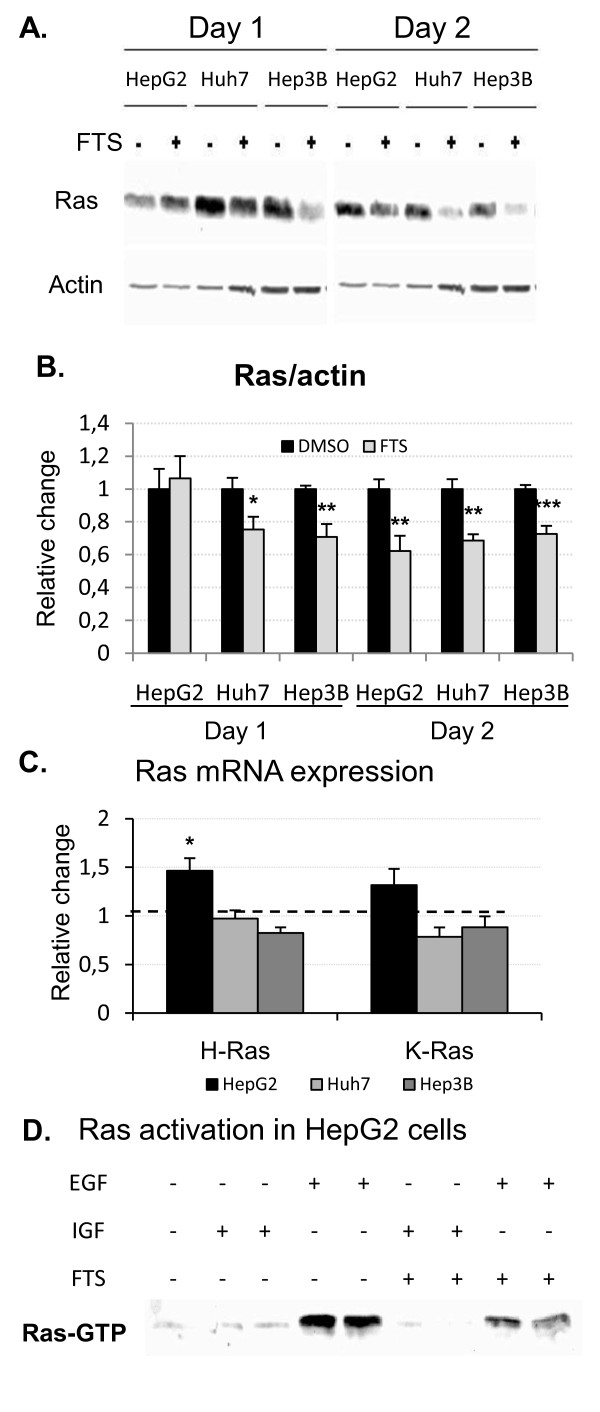
**Impact of salirasib on ras expression and activation**. A. Representative Western blots of ras after one and two days of treatment. B. Western blot of the independent experiments were quantified by densitometry and then normalized to their respective β-actin (n = 6 per group) Data are finally expressed as relative change compared to the control (DMSO) group arbitrarily set at 1. C. Quantification of H- and K-Ras mRNA expression by quantitative PCR after one day of treatment with salirasib (n = 6 per group). Data of the independent experiments were first normalized to RPL19 (internal standard) and then expressed as relative change versus control (DMSO) set arbitrarily at 1 (dotted line) and are presented as mean ± SEM. * *P *< 0.05, ** *P *< 0.01 and *** *P *< 0.001 in treated groups versus control group. D. Representative result of ras pull-down assays performed in 2 independent experiments in HepG2 cells stimulated with either EGF or IGF2 and treated with DMSO or 150 μM salirasib for 2 hours prior to stimulation.

As salirasib is known to inhibit ras activity and to promote its degradation, we studied its impact on ras expression in FBS-cultured cells by Western blot and quantitative PCR [13]. Exposure of cells to salirasib for 48 hours decreased ras protein expression in all three cell lines. Moreover this was already detectable after 24 hours in Huh7 and Hep3B but not in HepG2 cells (figure 6A-B). Decreased ras protein levels were not related to repression of H-ras or K-ras gene transcription (figure 6C).

To further confirm the impact of salirasib on ras activation, a ras pull-down assay was performed in HepG2 cells stimulated with EGF or IGF2 after 2 hours of incubation with DMSO or salirasib (figure 6D). EGF induced a strong activation of ras compared to serum-starved cells whereas activated ras after IGF2 stimulation remained at the level of unstimulated cells. Salirasib strongly reduced EGF-induced ras activation, and also decreased the expression of activated ras observed in IGF2-stimulated cells.

### The growth inhibitory effect of salirasib in HCC cell lines is associated with mTOR inhibition independent of ERK or Akt activation

In order to evaluate the impact of salirasib on ras mediated signaling, changes in the phosphorylation levels of key proteins were determined upon EGF- and IGF2-stimulation in our cell lines. ERK phosphorylation was used to monitor Raf/MAPK pathway activation, Akt and glycogen synthase kinase-3β (GSK3β) phosphorylation were used to measure PI3K/Akt activation, and p70 S6 kinase (p70) was used as a surrogate marker for mTOR activation.

**Figure 7:**
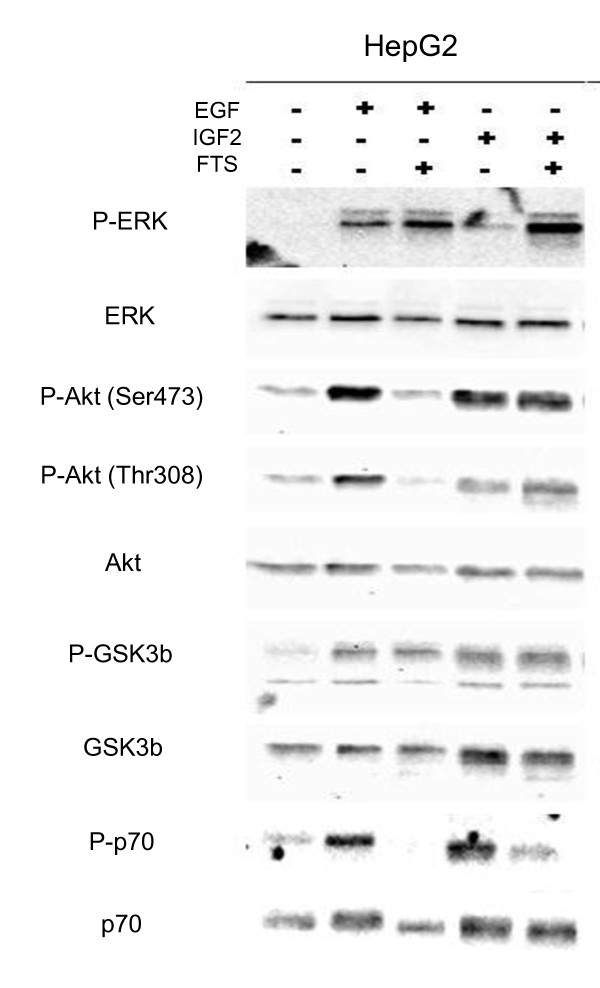
**Impact of salirasib on raf/MEK/ERK and PI3K/Akt/mTOR pathways in HepG2 cells**. Representative Western blots of 3 independent experiments of ERK, Akt, GSK3β, p70, and their phosphorylated counterparts in HepG2 cells stimulated with either EGF or IGF2 and treated with DMSO or 150 μM salirasib for 2 hours prior to stimulation Similar changes have been observed in the other cell lines (see supplementary figures 2 and 3).

In all 3 cell lines, EGF stimulation elicited a marked increase in ERK phosphorylation and preincubation with salirasib failed to reduce ERK phosphorylation (figure 7, additional file 4 - figure S2 and additional file 5 - figure S3). IGF2 stimulation did not induce ERK phosphorylation compared to controls, and treatment with salirasib prior to IGF2 increased phospho-ERK expression in HepG2 and Hep3B cells but not in Huh7 cells compared with controls and untreated IGF-stimulated cells (figure 7, additional file 4 - figure S2 and additional file 5 - figure S3).

The impact of treatment on Akt phosphorylation was dependent upon the cell line and culture condition. EGF induced Akt phosphorylation at Thr308 and Ser473 in all three cell lines. Pre-treatment with salirasib strongly reduced EGF-induced Akt phosphorylation in HepG2 cells (figure 7), but not in Hep3B or Huh7 cells (additional file 4 - figure S2 and additional file 5 - figure S3). IGF2 stimulated Akt phosphorylation in HepG2 and Hep3B cells that was not affected by pre-treatment with salirasib. By contrast, IGF2 did not increase Akt phosphorylation over controls in Huh7 cells but pre-treatment with salirasib induced Akt phosphorylation compared to controls as well as untreated IGF2 stimulated cells (additional file 4 - figure S2).

Variations in GSK3β phosphorylation levels paralleled those of Akt (figure 7).

Phosphorylation of p70 was low in unstimulated HepG2 and Hep3B cells but high in Huh7 cells. EGF stimulation induced phosphorylation of p70 in HepG2 and Hep3B, and to a lesser extent in Huh7 cells. IGF2 stimulation induced p70 phosphorylation in HepG2 and Hep3B cells, but did not further increase phospho-p70 levels above the already high baseline expression in Huh7. Importantly, salirasib abrogated p70 phosphorylation whether induced by EGF or IGF2 in HepG2 and Hep3B cells and completely suppressed baseline phospho-p70 expression in IGF2-stimulated Huh7 cells.

### Salirasib inhibits tumour growth in a subcutaneous xenograft model

**Figure 8:**
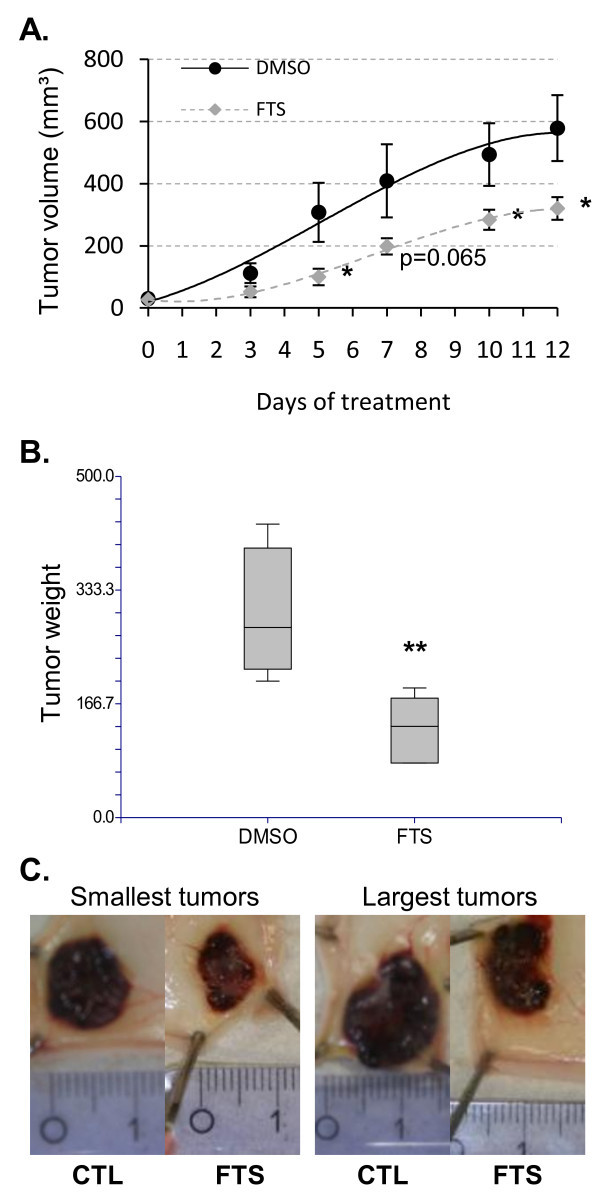
**Salirasib inhibits tumour growth in a subcutaneous xenograft model**. HepG2 cells were injected subcutaneously in the lower right flank of female NMRI nude mice. When palpable tumors were established, mice were treated with 10 mg/kg/day i.p. salirasib or vehicle for 12 days. A. Tumour volume was assessed three times per week as described in 'Materials and Methods'. Data are presented as mean ± SEM. B. At the end of the treatment period, animals were sacrificed and tumour weights were recorded. Boxplot of mean tumor volume at the time of sacrifice. C. Photographs of the smallest and largest tumours in the control group (CTL) and the FTS group (salirasib). * *P *< 0.05 ** *P *< 0.01 in treated group (n = 6) versus control group (n = 4).

Finally, we assessed the *in vivo *antitumor activity of salirasib in a subcutaneous xenograft model of HepG2 cells in nude mice. From 5 days of treatment onwards, salirasib induced a statistically significant decrease in tumour volume (figure 8A). After 12 days of salirasib treatment, the mean tumour weight was 131.7 ± 18.9 mg compared with 297.5 ± 48.2 mg in the control group (vehicle), indicating that salirasib reduced tumour growth by 56 percent (figure 8B). Moreover, no overlap in tumour weight was observed between the control and the treatment groups, meaning that even the smallest tumour in the control group remained larger than the biggest tumour in the treatment group (figure 8C). Animals remained well throughout the entire experiment and no weight loss was observed upon treatment, suggesting that salirasib was well tolerated at this dose regimen (data not shown).

## Discussion

Ras and mTOR are regarded as relevant therapeutic targets in HCC [9,10]. In this study, we report for the first time the effect of salirasib, a novel prenylcysteine analogue inhibiting cell growth in three human HCC cell lines through interference with ras and mTOR. Even more importantly, salirasib was able to inhibit both EGF- and IGF-induced proliferation in human HCC cell lines, potentially reducing the possibility for escape mechanisms related to activation of one growth factor pathway in response to the inhibition of the other one. Although IC_50 _were similar after three days of treatment in the three tested cell lines, time-course experiments suggests that Hep3B cells are the most sensitive to salirasib among the three tested cell lines, while Huh7 cells are more resistant. Importantly, our results also demonstrate that on the long-term salirasib treatment is effective at doses far below the estimated IC_50_.

The growth inhibitory effect is mainly mediated by inhibition of cell proliferation, which is observed in the three tested cell lines to a similar extent. This reduction of proliferation is associated with a profound modulation of the expression of cell cycle mediators. Cyclin A expression was strongly decreased in HepG2 and Huh7, and to a lesser extent in Hep3B. In the latter however, the cell cycle machinery disruption became clearly evident on the level of cyclin D1, the expression of which was almost completely abrogated upon treatment. In the two more sensitive cell lines, HepG2 and Hep3B, expression of the cell cycle inhibitors p21 and p27 was increased, reaching the highest magnitude in the most sensitive Hep3B cells. These observations partially mirror the impact of activated K-ras on the cell cycle, which is known to upregulate cyclin A and cyclin D, and to downregulate p27 [20]. On the other hand, mTOR inhibitors are known to induce a G1/S cell cycle arrest through an increase in p27 and a decrease in cyclin D [24] and cyclin A [25]. Thus, the impact of salirasib on cell proliferation might be due to a combination of both previously described effects of this compound, i.e. ras inhibition and mTOR inhibition [12,14].

On the other hand, apoptosis also contributes to the growth inhibitory effect of salirasib, and the relative resistance of Huh7 compared to the two other cell lines might be due to the absence of apoptosis induction upon treatment in these cells. However, the contribution of apoptosis seems to be less prominent than the anti-proliferative action of salirasib, at least under our experimental conditions. Indeed, caspase activation is more pronounced in HepG2 cells than in the more sensitive Hep3B cells. In addition, in these latter cells, no apoptosis induction could be observed at 50 μM or 100 μM salirasib, although these doses already induce a dramatic decrease in cell counts over time.

Nevertheless, high dose salirasib elicited caspase-3/7 activation in two cell lines that might at least partially be mediated by the mitochondrial apoptotic pathway. Apoptosis could have been caused in our cells by down-regulation of survivin, as salirasib has been shown to reduce survivin expression in glioblastoma cells [26], which was sufficient to elicit apoptosis. In addition, survivin down-regulation by antisense oligonucleotides has been shown to inhibit cell growth and to induce apoptosis in several cell lines, including HepG2 [27,28]. However, it was also repressed in the apoptosis-resistant Huh7 cells, suggesting that additional events are required to trigger cell death. Our results also suggest that salirasib might sensitize the cells to death receptor-induced apoptosis through up-regulation of the TRAIL receptors DR4 and DR5 in HepG2 and Hep3B cells, along with increased Fas expression in HepG2 cells and TNFα induction in Hep3B cells. Fas and TRAIL receptor upregulation alone might, however, not be sufficient to induce a major impact in vitro for their ligands, FasL and TRAIL, are mainly expressed on immune cells [29], which are not present in monocultures. Nevertheless, up-regulation of death receptors on tumor cells by treatments like salirasib and interaction with their respective ligands on immune cells could be of major importance in vivo, further potentiating the anti-tumor effect of salirasib.

Growth inhibition effects of salirasib are p53-independent as salirasib affect in a similar fashion HepG2 (p53 wild type) and Hep3B (p53 null) cells. This is further substantiated by the decrease in p53 expression observed after 2 days of treatment in HepG2 cells. This aspect could be clinically relevant, because most human HCC harbor defective p53 function [1]. A treatment strongly depending upon p53 activation could thus be less effective in these tumors. Our results contrast with a previous report of increased p53 function in colon cancer cells in response to salirasib [21]. However, p53 downregulation is compatible with ras inhibition, because K-ras activation is known to induce p53 up-regulation [20]. This lack of p53 upregulation in our study could be related to the absence of ERK inhibition upon treatment. Indeed, in HepG2 cells, ERK is a major activator of Mdm2, which is responsible for p53 degradation [30].

Total Ras protein expression was reduced in the three tested cell lines after 2 days of treatment, while Ras mRNA levels remained stable. In addition, salirasib reduced the expression of active GTP-bound Ras in HepG2 cells stimulated with EGF. These observations indicate an increase in ras protein degradation, which is consistent with the postulated mechanism of action of salirasib, involving the dislodgement of ras from the cell membrane followed by a cytosolic degradation [13]. Surprisingly, salirasib was unable to inhibit neither ERK nor Akt phosphorylation. On the contrary, it even tended to increase their phosphorylation levels, which could be due to a strong inhibition of p70 and to the consequent relief of a negative feedback loop affecting ERK and Akt [31].

Importantly, p70 phosphorylation was abrogated upon treatment in all cell lines when stimulated with EGF, which occurred without concomitant inhibition of ERK or Akt, both of which are known to activate mTOR. Moreover, salirasib also efficiently reduced p70 phosphorylation in all cell lines upon IGF2 stimulation, a situation where stimulation of the Akt-mTOR axis is independent of ras activation [8]. Indeed, no ras activation above baseline levels was observed in HepG2 cells stimulated with IGF2, and IGF2 did not induce ERK phosphorylation in any of the tested cell lines. Altogether, these data suggest that salirasib induced inhibition of mTOR in HCC cells occurs, at least in part, independently of ras, and thus point to a direct inhibitory effect on the mTOR complex 1, confirming earlier observations [14].

Nonetheless, it should not be concluded that the growth inhibitory effect that is observed in HCC cell lines solely relies on mTOR inhibition, as other unexplored ras mediators could be affected. Although, both ras and mTOR inhibition taken separately could explain the decrease in cyclin A and the increase in p27 levels, it is worth to note that these changes parallel the downregulation of ras in HepG2 and Hep3B cells.

Finally, we show that salirasib inhibits tumour growth *in vivo *in a subcutaneous xenograft model at a well tolerated dose. As salirasib is metabolized in the liver by cytochrome P450 2C subfamily (Concordia Pharmaceutical Inc., personal communication), there might be some concern about its potential efficacy in this organ. With regard to maintaining its efficiency in the liver as a target organ, we have shown that low-dose of salirasib prevented tumour occurrence in a model of diethylnitrosamine-induced hepatocarcinogenesis [18], while others have shown an impact of low-dose salirasib on liver fibrosis both in the preventive and the curative settings [32,33]. Both observations confirm that salirasib remains active in the liver.

## Conclusions

Our results indicate that salirasib elicits a dose- and time-dependent growth inhibitory effect in human HCC cell lines, related to inhibition of both EGF- and IGF-induced cell proliferation, and to a lesser extent to induction of apoptosis. This effect is linked with ras and mTOR inhibition, while ERK and Akt remained activated. Furthermore, we show that salirasib also exhibits anti-tumor activity *in vivo *in a mouse subcutaneous xenograft model. Our group has also previously described that salirasib prevents the development of preneoplastic liver foci in an animal model of diethylnitrosamine-induced hepatocarcinogenesis [18]. These results in human HCC cell lines, along with our previous observation of tumor prevention *in vivo *provide a rationale for testing salirasib in human HCC. Furthermore, investigation of combination therapies of salirasib and inhibitors of the raf/MEK/ERK pathway, the PI3K/Akt pathway, as well as combination with apoptosis-inducing treatments such as conventional chemotherapy or TRAIL-agonists are warranted in order to try to further improve the antitumor effect of salirasib.

## Abbreviations

BrdU: Bromodeoxyuridine; BSA: bovine serum albumin; cFLIP: cellular FLICE-like inhibitory protein; DMSO: dimethylsulfoxyde; DR: death receptor; EGF: epidermal growth factor; EGFR: EGF receptor; ERK: extracellular signal-regulated kinase; FBS: fetal bovine serum; FTS: farnesylthiosalicylic acid, salirasib; GSK3β: glycogen synthase kinase-3; HCC: hepatocellular carcinoma; IGF: insulin-like growth factor; IGF-1R: type 1 IGF receptor; i.p: intraperitoneal; mTOR: mammalian target of Rapamycin; p70: p70 S6 kinase; PBS: phosphate-buffered saline; PI3K: phosphoinositide 3-kinase; TNF: tumor necrosis factor; TRAIL: TNF-related apoptosis inducing ligand

## Competing interests

The authors declare that they have no competing interests.

## Authors' contributions

NC was involved in designing and performing the experiments, carried out analysis of data and drafted the manuscript. CD and VL carried out experiments. YH and IL participated in the design of the experiments. PS conceived the study, participated in its design and coordination, and helped to draft the manuscript. All authors read and approved the final manuscript.

## Acknowledgements

The authors are grateful to Martine Petit for expert technical assistance. This work was supported by Saint Luc Foundation and University FSR grants to PS. NC is the recipient of Saint Luc Foundation and FSR fellowships. IL is a FRS-FNRS research associate.

## Supplementary Material

Additional file 1 Supplementary table 1 - Western blot antibodies and working condition Click here for file

Additional file 2 Supplementary table 2 - Quantitative PCR primers Click here for file

Additional file 3 **Supplementary figure 1 - Salirasib modulates cell cycle distribution**. HepG2 (upper row), Huh7 (middle row), and Hep3B (lower row) cells were seeded in 6-well plates and incubated with DMSO (control) or 150 μM salirasib. Cell cycle distribution was assessed after 3 days of treatment. Data are presented as mean percent of cells in Sub-G0, G0/G1, S, and G2/M phases. * *P *< 0.05, ** *P *< 0.01 and *** *P *< 0.001 in treated groups versus control group (n = 6 in both groups). Click here for file

Additional file 4 **Supplementary figure 2 - Impact of salirasib on raf/MEK/ERK and PI3K/Akt/mTOR pathways in Huh7 cells**. Representative Western blots of 3 independent experiments of ERK, Akt, GSK3β, p70, and their phosphorylated counterparts in Huh7 cells stimulated with either EGF or IGF2 and treated with DMSO or 150 μM salirasib for 2 hours prior to stimulation. Click here for file

Additional file 5 **Supplementary figure 3 - Impact of salirasib on raf/MEK/ERK and PI3K/Akt/mTOR pathways in Hep3B cells**. Representative Western blots of 3 independent experiments of ERK, Akt, GSK3β, p70, and their phosphorylated counterparts in Hep3B cells stimulated with either EGF or IGF2 and treated with DMSO or 150 μM salirasib for 2 hours prior to stimulation. Click here for file
