# Supplementary material for: Salirasib inhibits the growth of hepatocarcinoma cell lines in vitro and tumor growth in vivo through ras and mTOR inhibition
Source: Mol Cancer. 2010 Sep 22;9:256. doi: 10.1186/1476-4598-9-256 (PMC2955616; doi:10.1186/1476-4598-9-256)
Supplement: Additional file 1 — Supplementary table 1 - Western blot antibodies and working condition [file 1476-4598-9-256-S1.PDF]

Supplementary table 1 - Western blot antibodies and working conditions

Antibody	Ref #	Provider	Dilution	Incubation time	Temperature
Anti-cyclin A	Sc-596	Santa Cruz	1/1000	Overnight	4°C
Anti-cyclin D1	Sc-8396	Santa Cruz	1/500	Overnight	4°C
Anti-cyclin E	Sc-481	Santa Cruz	1/4000	Overnight	4°C
Anti-Cdk2	Sc-163	Santa Cruz	1/2000	1h	Room temperature
Anti-Cdk4	Sc-260	Santa Cruz	1/2000	1h	Room temperature
Anti-p27	Sc-528	Santa Cruz	1/500	Overnight	4°C
Anti-p53	Sc-6243	Santa Cruz	1/1000	1h	Room temperature
Anti-cytochrome c	Sc-13156	Santa Cruz	1/1000	Overnight	4°C
Anti-Bcl-X_l	Sc-8392	Santa Cruz	1/500	Overnight	4°C
Anti-Mcl1	Sc-819	Santa Cruz	1/500	Overnight	4°C
Anti-ras	05-516	Millipore	1/1000	Overnight	4°C
Anti-ERK	9107	Cell Signaling	1/500	1h	Room temperature
Anti-phospho-ERK (Thr202/Tyr204)	9101	Cell Signaling	1/1000	Overnight	4°C

Anti-Akt	610861	BD Biosciences	1/2000	1h	Room temperature
Anti-phospho-Akt (Thr308)	2965	Cell Signaling	1/1000	Overnight	4°C
Anti-phospho-Akt (Ser473)	4060	Cell Signaling	1/1000	Overnight	4°C
Anti-GSK3β	9315	Cell Signaling	1/1000	Overnight	4°C
Anti-phospho-GSK3β (Ser9)	9336	Cell Signaling	1/1000	Overnight	4°C
Anti-p70	9202	Cell Signaling	1/1000	1h	Room temperature
Anti-phospho-p70 (Thr389)	9205	Cell Signaling	1/1000	Overnight	4°C
Anti-actin	A5441	Sigma	1/80000	1h	Room temperature
Anti-HSP90	610419	BD Biosciences	1/2500	1h	Room temperature
