# Supplementary material for: Salirasib inhibits the growth of hepatocarcinoma cell lines in vitro and tumor growth in vivo through ras and mTOR inhibition
Source: Mol Cancer. 2010 Sep 22;9:256. doi: 10.1186/1476-4598-9-256 (PMC2955616; doi:10.1186/1476-4598-9-256)
Supplement: Additional file 2 — Supplementary table 2 - Quantitative PCR primers [file 1476-4598-9-256-S2.PDF]

Supplementary table 2 - Quantitative PCR primers

Gene	Forward (5'-3')	Reverse (5'-3')	GeneBank accession #
p21	GCGGCAGACCAGCATGA	ATTAGGGCTTCCTCTTGGA GAAG	**NM_000389**
DR4	GGCTGAGGACAATGCTC ACA	TTGCTGCTCAGAGACGAAA GTG	**NM_0011271 83**
DR5	GACTCTGAGACAGTGCT TCGATGA	CCATGAGGCCCAACTTCCT	**NM_003842**
TNFα	GGAGAAGGGTGACCGA CTCA	TGCCCAGACTCGG CAAAG	**NM_000594**
Fas	CACTGTGACCCTTGCAC CAA	AAGTTAGATCTGGATCCTT CCTCTTTG	**NM_000043**
cFLIP	GTGTATGGTGTGGATCA GACTCACT	CATGAATCTCCCATGAACA TCCT	**NM_003844**
Survivin	CGAGGCTGGCTTCATCC A	CAACCGGACGAATGCTTTT T	**NM_001168**
H-ras	ACGGCATCCCCTACATC GA	ACCAACGTGTAGAAGGCAT CCT	**NM_005343**
K-ras	GCTGGTGGCGTAGGCAA GAG	CTCCTCTTGACCTGCTGTGT CG	**NM_033360**
RPL19	CAAGCGGATTCTCATGG AACA	TGGTCAGCCAGGAGCTTCT T	**NM_000981**
