# Supplementary figures and images for: Salirasib inhibits the growth of hepatocarcinoma cell lines in vitro and tumor growth in vivo through ras and mTOR inhibition
Source: Mol Cancer. 2010 Sep 22;9:256. doi: 10.1186/1476-4598-9-256 (PMC2955616; doi:10.1186/1476-4598-9-256)

# HepG2 (day 3)

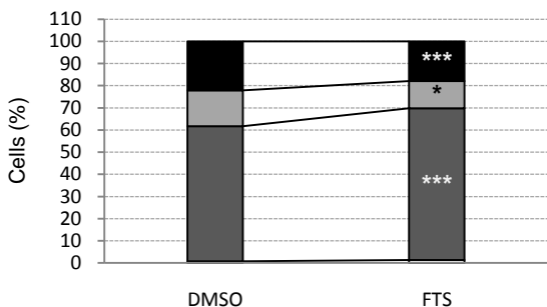

# Huh7 (day 3)

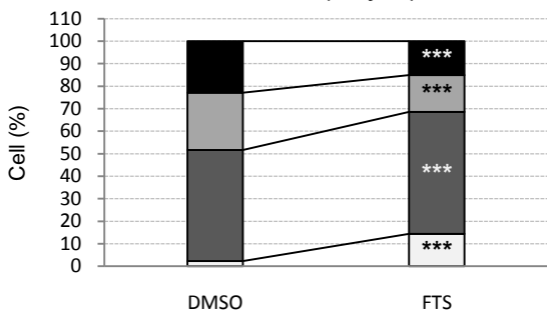

# Hep3B (day 3)

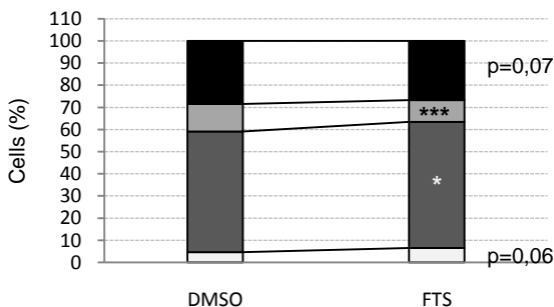

□ SubG0 ■ G0/G1 ■ S ■ G2/M

Supplement: Additional file 3 — Supplementary figure 1 - Salirasib modulates cell cycle distribution. HepG2 (upper row), Huh7 (middle row), and Hep3B (lower row) cells were seeded in 6-well plates and incubated with DMSO (control) or 150 μM salirasib. Cell cycle distribution was assessed after 3 days of treatment. Data are presented as mean percent of cells in Sub-G0, G0/G1, S, and G2/M phases. * P < 0.05, ** P < 0.01 and *** P < 0.001 in treated groups versus control group (n = 6 in both groups). [file 1476-4598-9-256-S3.PDF]

# Huh7

EGF  
IGF2  
FTS

| EGF | IGF2 | FTS |
|-----|------|-----|
| -   | -    | -   |
| +   | -    | -   |
| +   | -    | +   |
| -   | +    | -   |
| -   | +    | +   |

P-ERK

ERK

P-Akt (Ser473)

P-Akt (Thr308)

Akt

P-GSK3b

GSK3b

P-p70

p70

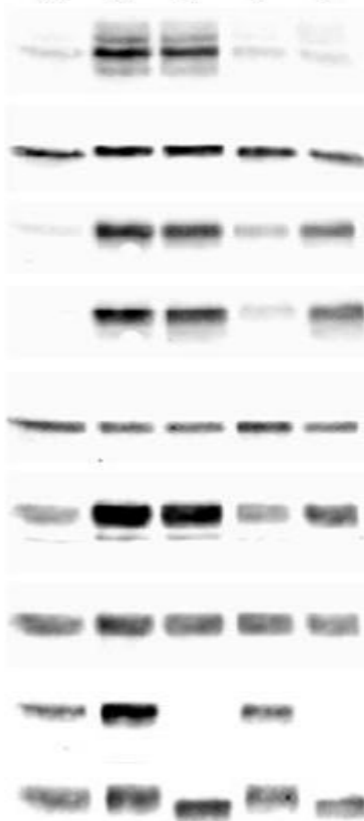

Supplement: Additional file 4 — Supplementary figure 2 - Impact of salirasib on raf/MEK/ERK and PI3K/Akt/mTOR pathways in Huh7 cells. Representative Western blots of 3 independent experiments of ERK, Akt, GSK3β, p70, and their phosphorylated counterparts in Huh7 cells stimulated with either EGF or IGF2 and treated with DMSO or 150 μM salirasib for 2 hours prior to stimulation. [file 1476-4598-9-256-S4.PDF]

# Hep3B

EGF  
IGF2  
FTS

|   |   |   |   |   |
|---|---|---|---|---|
| - | + | + | - | - |
| - | - | - | + | + |
| - | - | + | - | + |

P-ERK

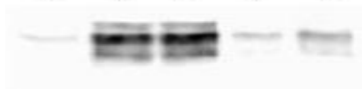

ERK

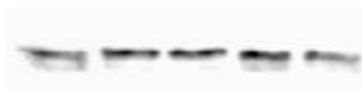

P-Akt (Ser473)

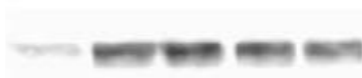

P-Akt (Thr308)

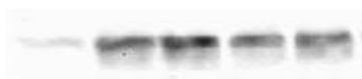

Akt

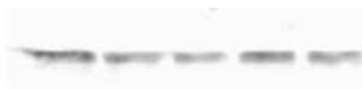

P-GSK3b

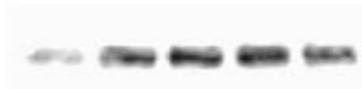

GSK3b

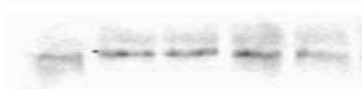

P-p70

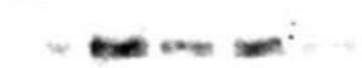

p70

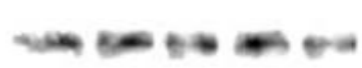

Supplement: Additional file 5 — Supplementary figure 3 - Impact of salirasib on raf/MEK/ERK and PI3K/Akt/mTOR pathways in Hep3B cells. Representative Western blots of 3 independent experiments of ERK, Akt, GSK3β, p70, and their phosphorylated counterparts in Hep3B cells stimulated with either EGF or IGF2 and treated with DMSO or 150 μM salirasib for 2 hours prior to stimulation. [file 1476-4598-9-256-S5.PDF]
